# Aloe vera for prevention of radiation-induced dermatitis: A systematic review and cumulative analysis of randomized controlled trials

**DOI:** 10.3389/fphar.2022.976698

**Published:** 2022-09-29

**Authors:** Tingting Wang, Jian Liao, Liying Zheng, Yi Zhou, Qianru Jin, Yanjing Wu

**Affiliations:** ^1^ Department of Integrated Traditional Chinese and Western Medicine, Taizhou Central Hospital (Taizhou University Hospital), Taizhou, Zhejiang, China; ^2^ Department of Nephrology, Jiaxing Hospital of Traditional Chinese Medicine, Jiaxing, Zhejiang, China; ^3^ Postgraduate Department, First Affiliated Hospital of Gannan Medical College, Ganzhou, China; ^4^ The 2nd Clinical Medical College, Zhejiang Chinese Medical University, Hangzhou, Zhejiang, China; ^5^ Second School of Clinical Medicine, Wenzhou Medical University, Wenzhou, China; ^6^ Department of Skin & Cosmetic, The Third Affiliated Hospital of Zhejiang Chinese Medical University, Hanzhou, Zhejiang, China

**Keywords:** aloe vera, radiation-induced dermatitis, systematic review, cumulative analysis, prevention

## Abstract

**Background:** Aloe vera were frequently reported to reduce the risk of radiation-induced dermatitis (RID), but the quantitative results from all the relevant studies were not presently available. This study sought to conduct a cumulative analysis to better clarify the preventive effects of aloe vera in RID.

**Methods:** MEDLINE (PubMed), Cochrane, EMBASE, PsychINFO, Web of Science, China National Knowledge Infrastructure (CNKI), and Wan Fang Database were utilized for identifying the eligible randomized controlled trials (RCTs) without language restrictions, up to March 2022. The pooled incidence of RID was conducted by the Relative risk (RR) with its 95% confidence interval (CI) through the STATA software under a random-effects model. This systematic review and cumulative analysis were registered on PROSPERO (ID: CRD42022335188).

**Results:** Fourteen RCTs met our predefined inclusion criteria, enrolling 1,572 participants (mean age: 46.5–56 years). The cumulative results revealed that patients pretreated with aloe vera were associated with a significantly lower risk of RID compared to those without aloe vera usage (RR = 0.76, 95% CI: 0.67–0.88, *p* < 0.001; heterogeneity: *I* ^*2*^ = 79.8%, *p* < 0.001). In the subgroup analysis, the pooled incidence of Grade 2–4, Grade 2, and Grade 3 RID was also dramatically lower in the group of aloe vera as compared to the placebo group [RR = 0.44 (0.27, 0.74), 0.58 (0.36, 0.94), and 0.27 (0.12, 0.59) in Grade 2–4, Grade 2, and Grade 3, respectively]. However, in regard to Grade 4 RID, the combined RR indicated that the incidence of RID was comparable between aloe vera and the control group (RR = 0.13, 95% CI: 0.02–1.01, *p* = 0.051; heterogeneity: *I* ^*2*^ = 0.0%, *p* = 0.741). The sensitivity analyses showed that there was no substantial change in the new pooled RR after eliminating anyone of the included study.

**Conclusion:** The current cumulative analysis revealed that patients pretreated with aloe vera were less likely to suffer from RID than the controls without using aloe vera. Based on this finding, the prophylactic application of aloe vera might significantly reduce the incidence of RID, especially in Grade 2 and Grade 3 RID. Further large-sample multicenter RCTs are still warranted to confirm these findings and for better clinical application.

## Introduction

Cutaneous disease is a global public health concern and brings immense health and economic burden on society because of its increasing prevalence. Since the worldwide population are rapidly ageing, the prevalence and the profile of skin diseases are growing, leading it to becoming a public health problem entailing an enormous socio-economic burden (Yew et al., 2022). Of the cutaneous manifestations of the disease, dermatitis has attracted great attention because of its global prevalence, and can be caused by endogenous and exogenous, including atopic dermatitis (AD), radiation dermatitis, contact dermatitis, and allergic contact dermatitis (Woo et al., 2019). It is difficult to determine the aetiological factors due to the similar skin changes of radiation dermatitis, atopic dermatitis, irritant dermatitis, and allergic contact dermatitis.

Approximately 50% of diagnosed cancer patients will receive radiotherapy alone or in combination with surgery or chemotherapy (Zhang et al., 2022). Radiation dermatitis is a common side effect of radiotherapy, affecting up to 95% of patients receiving radiation for cancer (Liao et al., 2019). As is well known, radiotherapy, a widely employed treatment for various cancers, inevitably involves exposing the skin to ionizing radiation. When the skin receives a relatively high dose of radiation, the epithelial cells of the skin can undergo a series of changes, which can lead to skin damage such as erythema dry or moist desquamation, and even ulceration or necrosis (Chitapanarux et al., 2019). Ionizing radiation contributes to DNA double-strand breaks by producing free radicals, and then causes acute inflammatory reactions in the skin (Kasmann et al., 2020; Tang et al., 2021). Additionally, progressive stenosis of the vasculature can be induced by radiation, leading to local ischemia and hypoxia, which exacerbates skin damage (Liao et al., 2019; Wang et al., 2021). The above studies indicate that the combined action of multiple factors results in the occurrence of radiation dermatitis. Although various treatments have been proposed to control dermatitis-associated symptoms of certain patients, including antihistamines and topical corticosteroids for atopic dermatitis, and steroidal, non-steroidal, and topical preparations and dressings for radiation dermatitis, several side effects remain, such as osteopenia and cataracts (Fallah et al., 2018; Choi et al., 2020; Umehara et al., 2020). In general, topical formulations, showing minimum side effects, are generally preferred. A previous study has demonstrated that the anionic polar phospholipid (APP) skin cream, a novel oil-in-water emulsion, has a preventive effect against radiation dermatitis (Merchant et al., 2007). Recently, mounting studies have shown that aloe vera can be used to treat dermatitis, including atopic dermatitis or radiation dermatitis (Richardson et al., 2005; Finberg et al., 2015).

Aloe vera (L.) Burm.f. (Aloe vera), a traditional Chinese medicine cataloged in the Pharmacopoeia of the People’s Republic of China, had been widely used traditionally for the treatment of constipation, wound healing, anorexia, and dyspepsia (Shi et al., 2021). Aloe vera contains aloin, emodin, polysaccharides, vitamins (A, C, E), amino acids, and other biologically active constituents, and is proven to have anti-inflammatory, anti-oxidant, immune modulation, and anti-tumorigenic effects (Ali and Wahbi, 2017). Merchant et al. (2007) reported that the APP skin cream common toxicity criteria (CTC) score, an acute radiation morbidity scoring criteria, was no larger than the aloe vera CTC score in all patients during radiation therapy. Furthermore, in 9 patients (20%), the aloe vera CTC score was larger than the APP skin cream CTC score, which suggested aloe vera was a safe and useful medicine for treating radiation therapy (Merchant et al., 2007). In contrast to this result, Ahmadloo et al. (2017) demonstrated that aloe vera had no association with the prevalence or severity of dermatitis.

Although the association between aloe vera and radiation dermatitis had been extensively studied, the effects of aloe vera in the prevention or treatment of radiation dermatitis were conflicting. Therefore the systematic review and cumulative analysis aim to provide a better understanding of the effectiveness and safety of aloe vera in the prevention or treatment of radiation dermatitis and to provide objective meaningful information to guide clinicians in clinical practice.

## Materials and methods

This systematic review and cumulative analysis were conducted according to the Preferred Reporting Items for Systematic Reviews and Meta-Analyses (PRISMA) guidelines (Supplementary Table S1). The detailed methods and information about this review could be found in the PROSPERO (ID: CRD42022335188, http://www.crd.york.ac.uk/PROSPERO).

### Data sources and searches

MEDLINE (PubMed), EMBASE (OVID), Cochrane Library databases, PsychINFO, Web of Science, China National Knowledge Infrastructure (CNKI), and Chinese Wan Fang Database were systematically searched for the relevant studies up to March 2022. The searching strategy was restricted to human subjects, while there were no language restrictions for the published articles. The searching keywords in PubMed database for English language was: {[“Aloe” (Mesh)] OR (Aloe vera)} AND {[“Dermatitis” (Mesh)] OR (Dermatitides)}.

### Assessment of radiation-induced dermatitis

The occurrence and severity of RID were diagnosed were graded using the Radiation Therapy Oncology Group (RTOG) standard, at the end of radiation therapy. According to the RTOG, the severity of RID would be 0 to 4 categories: grade 0, grade 1, grade 2, grade 3, and grade 4. High grade indicated greater severity of RID. Other assessments were also considered to be eligible, e.g., RID could be judged by a health care provider, as reported in the included studies.

### Inclusion criteria

The inclusion criteria for this study were listed as followed. 1) The study design should be conducted according to the patient, intervention, comparison, outcome, and study design (PICOS); The question guiding for this cumulative analysis was: Does aloe vera decrease the risk of RID? The compositions for the PICOS evidence were: cancer patients under radiation therapy (P); the usage of aloe vera (I); compared to those without aloe vera (C); the diagnosis of RID (O); any study designs (S). Furthermore, any trials presented with the relative risk (RR) and its 95% confidence intervals (CI) were also considered to be eligible.

### Exclusion criteria

In this study, we only included studies that reported human subjects, thus the *in vitro* and *in vivo* studies were excluded. Studies that failed to provide the data from the control participants were also excluded. Moreover, those publications belonging to review, comment, letter, and case reports could be also eliminated.

### Data extraction

The relevant information was independently extracted by two authors through a standardized data collection form. These data included the first authors’ names, country or region of the study, study design, the publication year, gender, types of cancer radiotherapy, dose of radiation (Gy), study period, the demographic and mean age of the participants with both groups, type of aloe vera, and assessment of RID.

### Risk of bias in the included studies

The risk of bias among the included studies was judged by Cochrane’s risk of the bias assessment tool. Based on the guideline of the Cochrane collaboration, the quality of the eligible studies was evaluated by the study design, methodology, and analysis. According to the different items, the included study could be assessed by a three-point scale: yes (low risk of bias), no (high risk of bias), and unclear (without specific statement).

### Statistical analyses

The cumulative RR with the corresponding 95% CI was applied to evaluate the strength of the association between aloe vera treatment and the risk of RID. Results derived from the combined RR with a two-tail *p* value < 0.05 were presumed as statistically significant. The *I* ^2^ statistic analysis was performed to identify the heterogeneity of a combined analysis. *I* ^2^ > 50% was considered to be substantial heterogeneity, while *p*-value of the *Q* test < 0.10 was assessed to be statistically significant. Since the presence of the different study designs as well as the study sample, a random effect model was applied when combining the RR. To detect the origin of the substantial heterogeneity, a sensitivity analysis was performed. Both Begg’s rank correlation test and Egger’s regression asymmetry test were used to assess the publication bias. In this cumulative analysis, the statistical analyses were performed with both the STATA (version 13.0, Stata Corp LP, College Station, Texas, United States) and the RevMan review management software (version 5.4).

## Results

### Literature search

**FIGURE 1:**
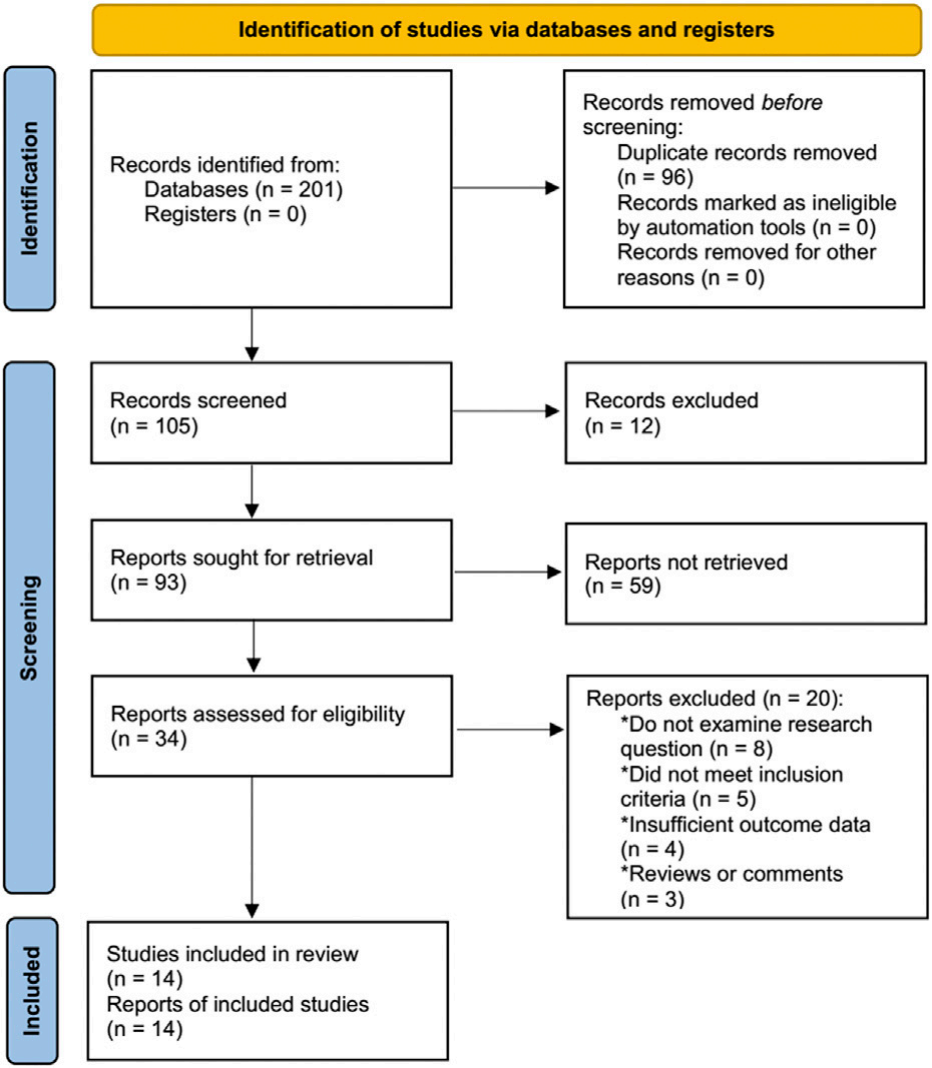
Flow chart of study selection. 201 studies were firstly identified and fourteen eligible studies were finally included after screening.

During the initial searching, 201 studies were detected. Among them, 91 publications were derived from PubMed and 37 publications were from the CNKI, while the remainder of 73 studies were detected from the EMBASE, Cochrane Library databases, PsychINFO, Web of Science, and Wan Fang Database. Based on a review of the titles and abstracts, 167 publications were eliminated due to duplicate and ineligible issues, leaving 34 potential studies for further full-text evaluation. Of which, 20 studies were eliminated for reasons, e.g., eight studies did not comply with the research question, five studies for failing to meet the inclusion criteria, four studies for insufficient outcome data, and three studies being to as a review or comment article. Finally, a total of fourteen studies (Williams et al., 1996; Deng and Feng, 2006; Liu et al., 2006; Yao et al., 2006; Liu, 2007; Hong et al., 2009; Wu and Dong, 2009; Haddad et al., 2013; Hoopfer et al., 2015; Wu et al., 2015; Ahmadloo et al., 2017; Zhong et al., 2017; Wang et al., 2018; Zhao et al., 2018) were considered to be eligible for further systematic review and quantitative analysis. The selection process of the fourteen publications was shown in Figure 1.

### Study characteristic

The descriptive characteristics of the fourteen eligible studies were summarized in Table 1. These included studies were all randomized control designed, and published between 1996 and 2018. Among the fourteen studies, one study was conducted in the United States, one in Saudi Arabia, two in Iran, and the reminder nine studies originated from China. A total of 1,572 participants (mean age: 46.5–56 years) were enrolled. The number of the aloe vera group and the control group was comparable (792 vs. 780). All these included studies provided the data on the total incidence of RID in both the study and the control group, while eight studies also provided the number of RID in each grade of dermatitis (Grade 0–4, Table 2). The types of cancer radiotherapy included nasopharyngeal carcinoma, breast cancer, and many other malignancies. In this study, both male and female participants were included. The study period ranged from 2 to 10 weeks. The single dose of radiation ranged from 40–74 Gy, and the total radiation quantity ranged from 2,000–7,000 Gy. The sample size in each included study ranged from 48 to 300 participants. The type of aloe vera included fresh, gel, cream, and lotion aloe. The assessment of dermatitis was mainly following the Radiation Therapy Oncology Group standard (RTOG), but the RID evaluation in one study was judged by a health care provider.

**TABLE 1:** Characteristics of the fourteen included studies.

Study	Study design	Gender	Study period	Types of cancer radiotherapy	Dose of Gy	Study group case/total (grade 1–4 dermatitis)	Placebo group case/total (grade 1–4 dermatitis)	Mean age (years)	Type of aloe vera	Assessment of dermatitis
Williams (18) 1996 United States	RCT-double-blinded	Female	NA	Breast cancer	45–60	88/97	94/97	NA	Gel	Judged by health care provider
Deng (19) 2006 China	RCT	Both sexes	NA	Nasopharyngeal carcinoma	60–72	4/35	10/32	S: 48.5	Fresh aloe vera	Radiation therapy oncology group standard
								C: 47		
Liu (20) 2006 China	RCT	Both sexes	NA	Various types of cancers	NA	25/40	36/40	NA	Fresh aloe vera	Radiation therapy oncology group standard
Yao (21) 2006 China	RCT	Both sexes	NA	Various types of cancers	Total dose: 3,000–7,000	20/34	31/35	S: 56	Fresh aloe vera	Radiation therapy oncology group standard
								C: 53		
Liu (22) 2007 China	RCT	Both sexes	NA	Various types of cancers	Total dose: 2000–7,000	2/30	20/30	30–78	Gel	Radiation therapy oncology group standard
Hong (23) 2009 China	RCT	Both sexes	NA	Nasopharyngeal carcinoma	7,000	50/50	50/50	S: 48	Fresh aloe vera	Radiation therapy oncology group standard
								C: 49		
Wu (24) 2009 China	RCT	Both sexes	2 weeks	Nasopharyngeal carcinoma and breast cancer	Nasopharyngeal carcinoma: 70; Breast cancer: 50	24/24	24/24	NA	Gel	Radiation therapy oncology group standard
Haddad (25) 2013 Iran	RCT	Both sexes	5 weeks	Various types of cancers	40–70	46/53	50/53	52 (21–78)	Lotion	Radiation therapy oncology group standard
Hoopfer (26) 2015 Saudi Arabia	RCT	Females	4 weeks	Breast cancer	45–50	7/81	5/77	NA	Aloe vera cream	Radiation therapy oncology group standard
Wu (27) 2015 China	RCT	Both sexes	7–10 days	Various types of cancers	NA	15/60	35/60	NA	Fresh aloe vera	Radiation therapy oncology group standard
Ahmadloo (28) 2017 Iran	RCT	Females	5 weeks	Breast cancer	50	45/50	47/50	S: 48.5	Gel	Radiation therapy oncology group standard
								C: 47		
Zhong (29) 2017 China	RCT	Both sexes	2–3 weeks	Nasopharyngeal carcinoma	50–74	52/153	97/147	S: 49.4	Fresh aloe vera	Radiation therapy oncology group standard
								C: 46.5		
Wang (30) 2018 China	RCT	Females	5–6 weeks	Breast cancer	50–60	33/36	34/36	52	Fresh aloe vera	Radiation therapy oncology group standard
Zhao (31) 2018 China	RCT	Both sexes	6–7 weeks	Nasopharyngeal carcinoma	50–60	29/49	39/49	S: 51	Gel	Radiation therapy oncology group standard
								C: 49.5		

Note: Study group = patients under radiotherapy pretreated with aloe vera (*n* = 792), Control group = placebo group, patients under radiotherapy receiving conventional treatments (*n* = 780), the incidence rate of Grade 1–4 dermatitis in the study group and the control group was 56% (440/792) vs. 73% (572/780), NA, not available.

**TABLE 2:** The incidence of grade 2–4, grade 2, grade 3, and grade 4 radiation-induced dermatitis in both the aloe vera group and the control group.

Study	Radiation induced dermatitis					
	Grade 0	Grade 1	Grade 2	Grade 3	Grade 4	Grade 2–4
Williams (18) 1996	S: 0/97	S: 0/97	S: 4/97	S: 0/97	S: 0/97	S: 4/97
	C: 0/97	C: 0/97	C: 5/97	C: 5/97	C: 0/97	C: 10/97
Deng (19) 2006	S: 15/35	S: 16/35	S: 7/35	S: 2/35	S: 0/35	S: 9/35
	C: 4/32	C: 11/32	C: 14/32	C: 9/32	C: 2/32	C: 25/32
Hong (23) 2009	S: 0/50	S: 50/50	S: 0/50	S: 0/50	S: 0/50	S: 0/50
	C: 0/50	C: 42/50	C: 5/50	C: 3/50	C: 0/50	C: 8/50
Wu (24) 2009	S: 0/24	S: 21/24	S: 3/24	S: 0/24	S: 0/24	S: 3/24
	C: 0/24	C: 10/24	C: 14/24	C: 0/24	C: 0/24	C: 14/24
Wu (27) 2015	S: 45/60	S: 10/60	S: 5/60	S: 0/60	S: 0/60	S: 5/60
	C: 25/60	C: 10/60	C: 15/60	C: 5/60	C: 5/60	C: 25/60
Ahmadloo (28) 2017	S: 5/50	S: 31/50	S: 12/50	S: 2/50	S: 0/50	S: 14/50
	C: 3/50	C: 36/50	C: 6/50	C: 5/50	C: 0/50	C: 11/50
Wang (30) 2018	S: 3/36	S: 16/36	S: 15/36	S: 2/36	S: 0/36	S: 17/36
	C: 2/36	C: 14/36	C: 17/36	C: 3/36	C: 0/36	C: 20/36
Zhao (31) 2018	S: 20/49	S: 18/49	S: 11/49	S: 0/49	S: 0/49	S: 11/49
	C: 10/49	C: 17/49	C: 21/49	C: 1/49	C: 0/49	C: 22/49

### Risk of bias assessment

**FIGURE 2:**
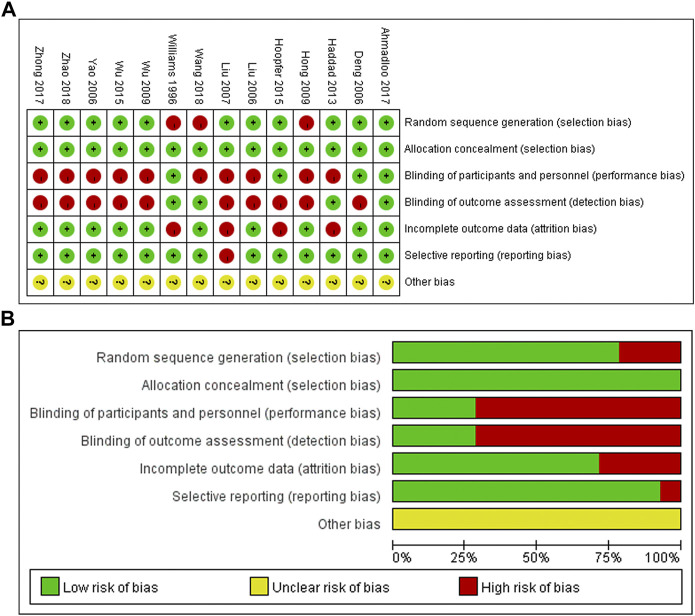
Risk of bias among the fourteen included studies **(A,B)**. The results turned out that most of the included studies (13/14, 93%) were with high risk of bias.

Among the fourteen included studies, only Ahmadloo et al. (2017)’s study was judged as low risk of bias, and the remaining thirteen studies were evaluated to present with a high risk of bias (Figure 2).

### Synthesis of results

**FIGURE 3:**
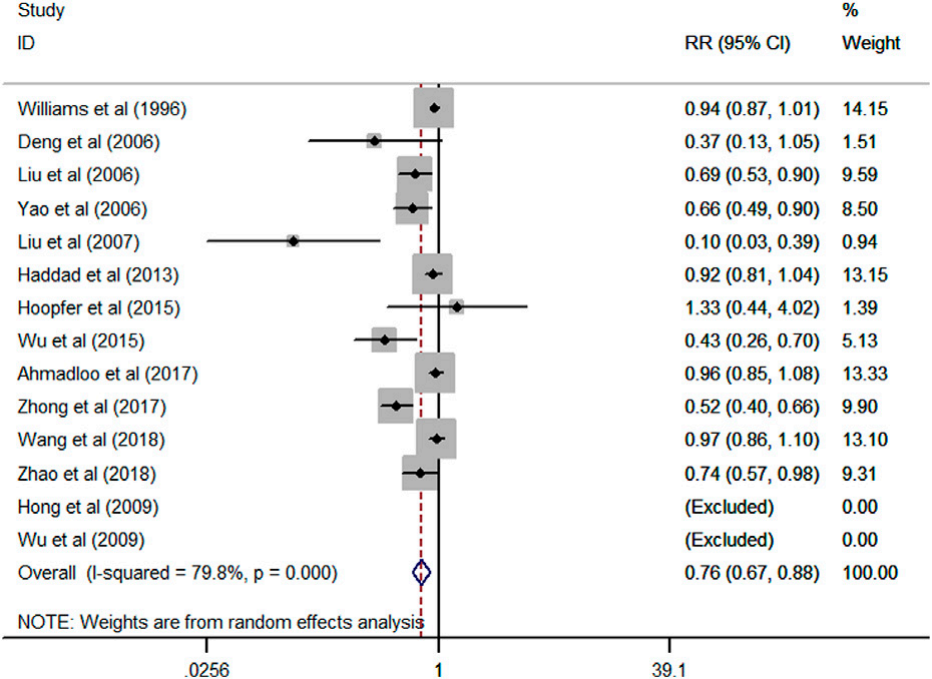
Forest plots of the cumulative analysis of the fourteen included studies on the association between pretreatment with aloe vera and the risk of radiation-induced dermatitis the incidence rate of RID in the study group and the control group: 56% (440/792) vs. 73% (572/780).

As shown in Figure 3, the cumulative RR from twelve included studies indicated that patients with radiotherapy pretreated with aloe vera were associated with a significantly lower risk of RID compared to the control group (those without aloe vera usage) (overall RID incidence: RR = 0.76, 95% CI: 0.67–0.88, *p* < 0.001; heterogeneity: *I* ^*2*^ = 79.8%, *p* < 0.001). Of note, Hong et al. and Wu et al. (2015)’s study showed the incidence of RID was 100% in both the study and the control group, resulting in these two studies could not be included in the statistical analyses with the STATA (displayed with “Excluded” in Figure 3).

**FIGURE 4:**
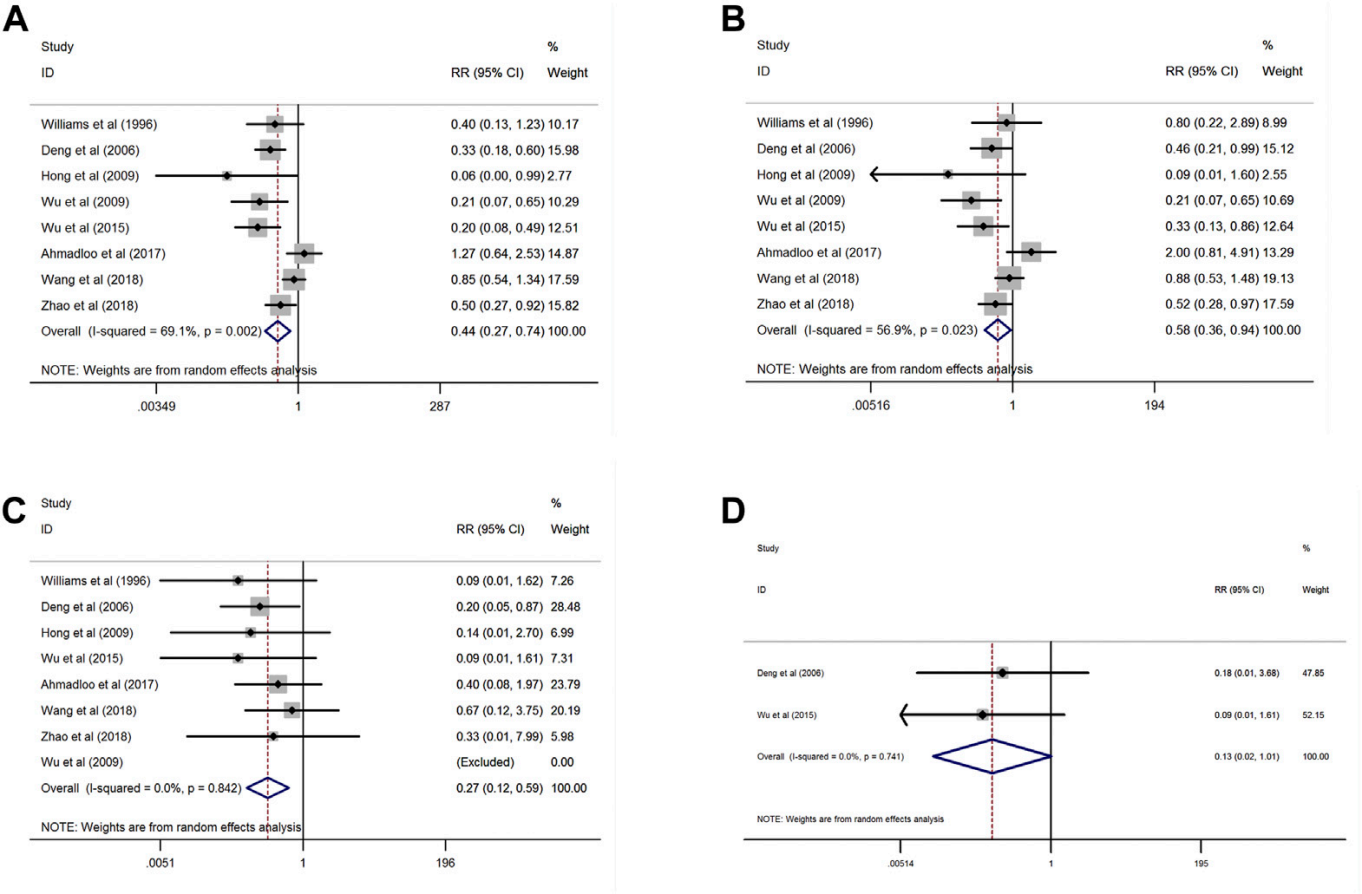
Forest plots of the cumulative analysis of the protective effects developed by aloe vera in Grade 2–4 **(A)**, Grade 2 **(B)**, Grade 3 **(C)**, and Grade 4 **(D)**radiation-induced dermatitis (study group: *n* = 401; control group: *n* = 398).

In the subgroup analysis, as presented in Figure 4A, the pooled incidence of Grade 2–4 RID was also dramatically lower in the group of aloe vera as compared to the placebo group (RR = 0.44, 95% CI: 0.27–0.74, *p* = 0.002; heterogeneity: *I* ^*2*^ = 69.1%, *p* = 0.002). When separated by each Grade of RID, the cumulative RR derived from eight included studies consistently suggested that pretreatment with aloe vera might significantly reduce the incidence of Grade 2 RID compared to the controls (RR = 0.58, 95% CI: 0.36–0.94, *p* = 0.026; heterogeneity: *I* ^*2*^ = 56.9%, *p* = 0.023, Figure 4B). In line with this finding, aloe vera usage also associated with a lower incidence of Grade 3 RID than the control group (RR = 0.27, 95%CI: 0.12–0.59, *p* = 0.001; heterogeneity: *I* ^*2*^ = 0.0%, *p* = 0.842, Figure 4C).

However, as displayed in Figure 3D, when restricted to Grade 4 RID, the quantitative synthesis of the results from two included studies revealed that the incidence of RID was comparable between aloe vera and the control group (RR = 0.13, 95%CI: 0.02–1.01, *p* = 0.051; heterogeneity: *I* ^*2*^ = 0.0%, *p* = 0.741, Figure 4D). This result indicated aloe vera exerted no protective effect against Grade 4 RID.

The above results indicated that patients pre-treated with aloe vera were associated with a significantly lower incidence of RID, including the overall incidence, Grade 2–4, Grade 2, and Grade 3 RID. Therein, the incidence of Grade 3 RID was the lowest under aloe vera treatment, decreasing the risk of RID by over 70%.

### Sensitivity analysis

**FIGURE 5:**
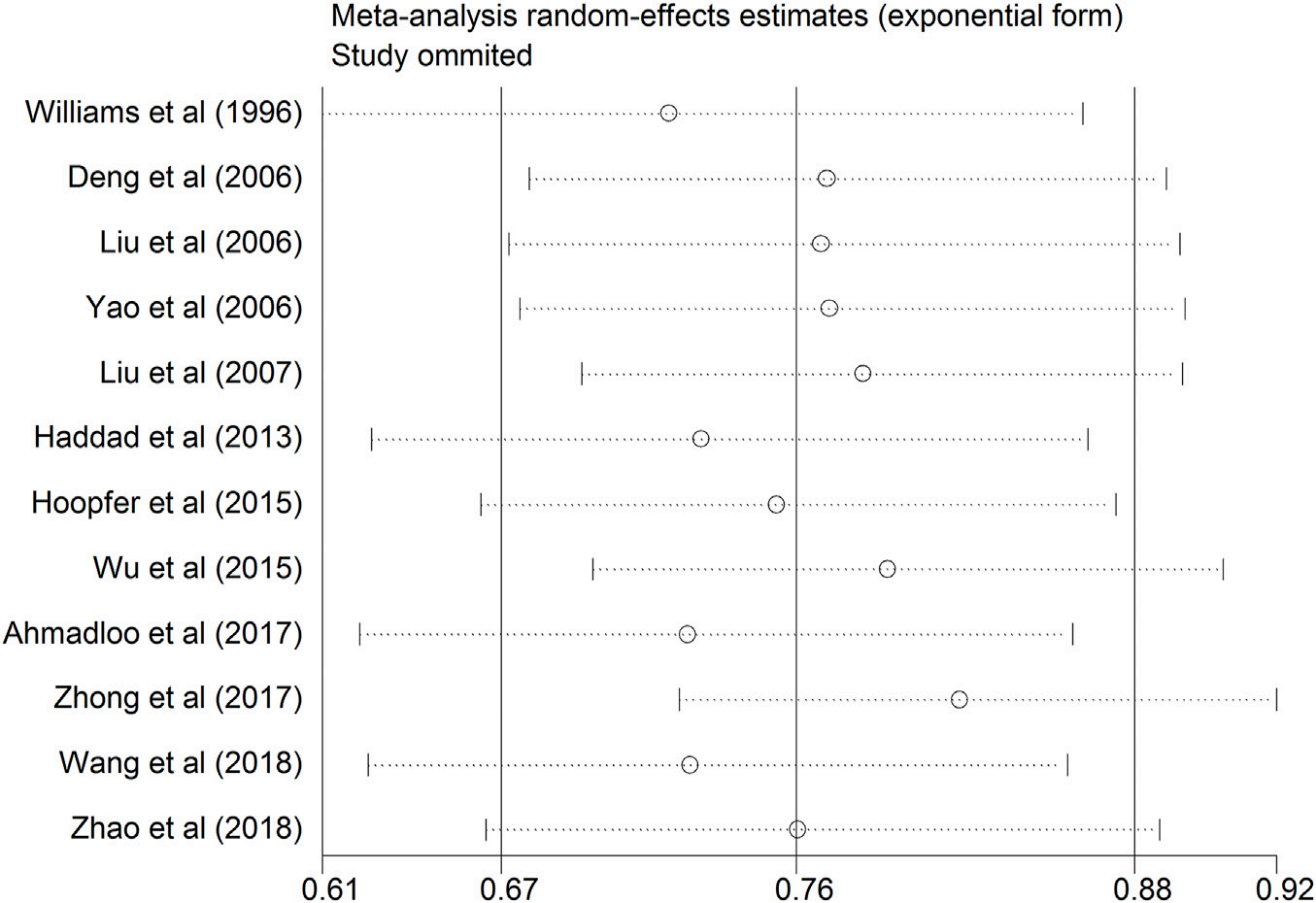
Sensitivity analysis after each study was excluded by turns with the STATA software.

To evaluate the effect of the single study on the combined quantitative synthesis of RR and the heterogeneity, a sensitivity analysis was properly conducted. As shown in Table 3; Figure 5, after removing anyone of the twelve eligible studies, no substantial difference was detected in the new cumulative RR [ranging from 0.73 (95%CI: 0.62–0.86) to 0.82 (95% CI: 0.72–0.92)]. Regarding the heterogeneity assessment, no remarkable change was observed in the newly generated heterogeneity after omitting any of the included studies (*I* ^2^ ranged from 71.6% to 81.5%, all *p* < 0.001). The above results revealed that no single study could dominate the combined RR and the heterogeneity.

**TABLE 3:** Sensitivity analyses after each study was excluded by turns.

Study omitted	RR (95% CI) for remainders	Heterogeneity (%)	
		*I* ^2^ (%)	*P*
Williams et al. (1996)	0.72 (0.61, 0.86)	80.4	<0.001
Deng and Feng (2006)	0.77 (0.68, 0.89)	80.6	<0.001
Liu et al. (2006)	0.77 (0.67, 0.89)	80.3	<0.001
Yao et al. (2006)	0.77 (0.67, 0.89)	80.3	<0.001
Liu (2007)	0.79 (0.69, 0.86)	77.5	<0.001
Haddad et al. (2013)	0.73 (0.62, 0.86)	81.5	<0.001
Hoopfer et al. (2015)	0.76 (0.66, 0.87)	81.4	<0.001
Wu et al. (2015)	0.79 (0.70, 0.91)	78.1	<0.001
Ahmadloo et al. (2017)	0.73 (0.62, 0.86)	80.9	<0.001
Zhong et al. (2017)	0.82 (0.72, 0.92)	71.6	<0.001
Wang et al. (2018)	0.73 (0.62, 0.85)	80.8	<0.001
Zhao et al. (2018)	0.76 (0.66, 0.88)	81.0	<0.001

Abbreviation: RR, relative risk; CI, confidence interval.

### Publication bias

**FIGURE 6:**
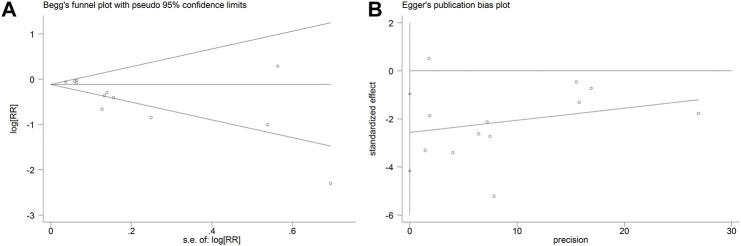
Begg’s rank correlation test **(A)** and Egger’s regression asymmetry test **(B)**.

The Begg’s rank correlation test suggested that there was no significant publication bias underlying this pooled analysis (Begg’s, *p* > |z| = 0.064) (Figure 6A). However, the Egger’s linear test showed a significant publication bias (Egger’s, *p* > |t| = 0.005, 95% CI: −4.17 to −0.96) (Figure 6B).

## Discussion

Acute dermatitis is a common adverse effect after radiation therapy, affecting over 90% of the patients. The severity of RID varied from erythema to ulcer and necrosis. There are currently numerous methods to prevent, reduce, and treat radiotherapy skin toxicities based on the experiences of the clinicians. Mounting evidence suggests that the traditional medicinal herbs and their extractives have an excellent effect on anti-RID, such as Jaungo, Plantago lanceolate folium, Heijiangdan Ointment, and aloe vera (Skalska-Kaminska et al., 2014; Kong et al., 2016; Yang et al., 2016). Aloe vera, an ancient traditional herbal, exhibits a remarkable anti-inflammatory effect in injury diseases. The first clinical study of aloe vera in the treatment of radiation dermatitis was first reported in 1935 (Williams et al., 1996). Since then, multiple studies have reported the effects of aloe vera in the prevention or treatment of radiation dermatitis. To better assess this role by undertaking a scientific analysis, we conducted a cumulative analysis to evaluate the effect of aloe vera in the prevention or treatment of radiation dermatitis. Fourteen RCTs were finally included for a pooled analysis, enrolling 1,572 participants (mean age: 46.5–56 years). According to the combined RR, patients pretreated with aloe vera substantially reduced the risk of Grade 1–4 RID by as much as 24% (RR = 0.76, 95% CI: 0.67–0.88, *p* < 0.001). In line with this finding, pretreatment with aloe vera was also associated with a significantly lower risk of Grade 2–4 RID when compared to the controls (RR = 0.44, 95% CI: 0.27–0.74, *p* = 0.002). In regard to each grade of RID, aloe vera could reduce the risk of Grade 2 by 42% (RR = 0.58, 95% CI: 0.36–0.94, *p* = 0.026) and Grade 3 by 73% (RR = 0.27, 95% CI: 0.12–0.59, *p* = 0.001). Subsequent sensitivity analyses revealed that no single study could dominate the overall combined RR. Therefore, the present evidence was robust.

As reported, aloe vera is found to contain over 70 potentially active constituents, e.g., vitamins, enzymes, salicylic acids, and amino acids (Surjushe et al., 2008). In 1995, the RTOG institutes showed that aloe vera could be applied as one of the prophylactic treatments for RID (Haddad et al., 2013). Although aloe vera had been already demonstrated to be preventive against radiation dermatitis in the present meta-analysis and some other studies, the specific mechanism for this effect had not been well understood. The pathogenesis of aloe vera is multifactorial, including anti-oxidant activity, anti-inflammatory effects, and the inhibition of vasoconstriction.

It was known that ionizing radiation is the direct cause of radiation dermatitis. DNA damage is considered to be the direct consequence of high dose ionizing radiation. Ionizing radiation had been shown to induce DNA damage and cell death (Ghosh and Ghosh, 2021). Meanwhile, ionizing radiation can amplify this effect by promoting the production of reactive oxygen species (ROS) and nitric oxide (NO) (Wilson et al., 2019; Kma and Baruah, 2022). It was reported that irradiated cells produced several enzymes by releasing inflammatory cytokines and growth factors such as NADPH oxidase, cyclooxygenase-2 (COX-2), and inducible nitric oxide synthesize (iNOS), which stimulated ROS and NO production (Abbaszadeh et al., 2017; Mortezaee et al., 2019; Chen et al., 2020). In addition, superoxide anions induced by the electron transfer chains of mitochondria enhanced intracellular oxidative stress (Brand, 2016). Also, free radicals, the intracellular signals, significantly potentiate the inflammatory response (Ramos-Tovar and Muriel, 2020). Importantly, oxidative stress and inflammation play important roles in radiation dermatitis (Yang et al., 2016; Sheng et al., 2019). Popanda et al. (2003) reported that several DNA damages were observed and DNA repair capacity was severely reduced in patients with radiation dermatitis. Another study showed that 18β-glycyrrhetinic acid significantly ameliorated radiation-induced skin damage by inhibiting NADPH oxidase activity and ROS production, consequently suppressing pro-inflammatory cytokine production (Su et al., 2018). Previous studies demonstrated that aloe vera had anti-inflammatory and anti-oxidative effects on various diseases such as peptic ulcers, colitis, and skin wounds (Akaberi et al., 2016; Hong et al., 2018). The results are in line with those of other studies. Goyal and Gehlot (2009) reported that aloe vera promoted the activities of both superoxide dismutase (SOD) and catalase, which could be beneficial to promoting free radical scavenging capacity, and consequently reduced the intensity of radiation dermatitis. Furthermore, aloe vera was effective in scavenging ROS and protecting DNA (Goyal and Gehlot, 2009). Haddad et al. (2013) demonstrated that the prophylactic use of aloe vera significantly reduced the intensity of radiation dermatitis. Similarly, Rao et al. (2017) also reported that the application of aloe vera decreased the incidence of Grade 1, 2, and 3 radiation dermatitis in head and neck cancer. Based on above evidence, aloe vera may mitigate the grade of radiation dermatitis by exerting anti-inflammatory and anti-oxidative effects.

Vascular damage is associated with the pathogenesis of radiation dermatitis (Brown and Rzucidlo, 2011). Additionally, aloe vera had been shown to protect against vascular damage. Soria et al. (2019) reported that immediately after radiation, vascular damage was observed in the brain. In line with the aforementioned studies, a result by Rong et al. (2019) indicated that vascular damage to the skin was rapidly induced in response to radiation. Human fetal skin-derived stem cells (hFSSC) is served as a preferable source for skin tissue regeneration. It has been reported that the hFSSC secretome significantly reduces the intensity of radiation dermatitis by enhancing angiogenesis through up-regulation of vascular endothelial growth factor (VEGF), placental growth factor (PLGF), and transforming growth factor β3 (TGF-β3) (Rong et al., 2019). Aloe vera also had been confirmed to infiltrate into the skin tissue and promote wound healing by improving angiogenesis (Tarameshloo et al., 2012). Consistent with this result, Ali et al. (2021) reported that aloe vera improved angiogenesis by promoting the expression of VEGF in the skin, which ameliorated skin wound healing. Thus, the efficacy of aloe vera on radiation dermatitis may be explained by the proangiogenic effect of aloe vera. Further studies are needed to evaluate the prevention efficacy and potential action mechanism of aloe vera in radiation dermatitis.

As far as we know, this is the first study to quantify the protective effects of aloe vera on RID through a cumulative analysis. Multiple strengths should be acknowledged in the present study. This is a meta-analysis of all the available studies with a larger sample with an evidence-based etiological theory. As a result, the present evidence is creditable for clinical practice. Nevertheless, several inherent limitations could not be ignored when interpreting this data. First, substantial heterogeneities existed in a combined analysis. In general, different study designs, sample sizes, ages of the participants, geographical areas, and confounding factors could all be partly responsible for this remarkable heterogeneity. Second, we only included those studies that were published in English and Chinese, which may induce a selection bias. Third, different ages and various types of cancers were found in these included studies, which may downgrade the evidence of this study. Thus, caution is warranted when interpreting this study. Besides, additional well-designed RCTs with large samples are still warranted to further illuminate the effects of radioprotection developed by aloe vera.

## Conclusion

Based on the current evidence, pre-treated with aloe vera was reinforced to significantly reduce the incidence of RID as compared with the conventional treatment. Aloe vera could be served as a standardized product in the prevention or treatment of RID in patients with malignancies, especially for breast cancer and nasopharyngeal carcinoma. On account of the small quantity of relevant RCTs, the conclusions should be interpreted carefully. In the near future, further multicenter RCTs with a large sample size would be required to confirm this finding.

## Supplementary material

SUPPLEMENTARY TABLE S1 PRISMA Checklist.

## Abbreviations

RID, radiation-induced dermatitis; CNKI, China National Knowledge Infrastructure; RCTs, randomized controlled trials; RR, Relative risk; CI, confidence interval; atopic AD, dermatitis; APP, anionic polar phospholipid; CTC, common toxicity criteria; PRISMA, Preferred Reporting Items for Systematic Reviews and Meta-Analyses; RTOG, Radiation Therapy Oncology Group; PICOS, patient, intervention, comparison, outcome, and study design; SOD, superoxide dismutase; VEGF, vascular endothelial growth factor; PLGF, placental growth factor; TGF-β3, transforming growth factor β3.

## References

[B1] Abbaszadeh A. Haddadi G. H. Haddadi Z. (2017). Melatonin role in ameliorating radiation-induced skin damage: From theory to practice (A review of literature). J. Biomed. Phys. Eng. 7 (2), 127–136. 28580334 PMC5447249

[B2] Ahmadloo N. Kadkhodaei B. Omidvari S. Mosalaei A. Ansari M. Nasrollahi H. (2017). Lack of prophylactic effects of aloe vera gel on radiation induced dermatitis in breast cancer patients. Asian Pac. J. Cancer Prev. 18 (4), 1139–1143. 10.22034/APJCP.2017.18.4.1139 28547955 PMC5494228

[B3] Akaberi M. Sobhani Z. Javadi B. Sahebkar A. Emami S. A. (2016). Therapeutic effects of aloe spp. in traditional and modern medicine: A review. Biomed. Pharmacother. 84, 759–772. 10.1016/j.biopha.2016.09.096 27716590

[B4] Ali F. Wajid N. Sarwar M. G. Qazi A. M. (2021). Oral administration of aloe vera ameliorates wound healing through improved angiogenesis and chemotaxis in sprague dawley rats. Curr. Pharm. Biotechnol. 22 (8), 1122–1128. 10.2174/1389201021999201001204345 33023442

[B5] Ali S. Wahbi W. (2017). The efficacy of aloe vera in management of oral lichen planus: a systematic review and meta-analysis. Oral Dis. 23 (7), 913–918. 10.1111/odi.12631 28029732

[B6] Brand M. D. (2016). Mitochondrial generation of superoxide and hydrogen peroxide as the source of mitochondrial redox signaling. Free Radic. Biol. Med. 100, 14–31. 10.1016/j.freeradbiomed.2016.04.001 27085844

[B7] Brown K. R. Rzucidlo E. (2011). Acute and chronic radiation injury. J. Vasc. Surg. 53, 15S–21S. 10.1016/j.jvs.2010.06.175 20843630

[B8] Chen S. Guo D. Lei B. Bi J. Yang H. (2020). Biglycan protects human neuroblastoma cells from nitric oxide-induced death by inhibiting AMPK-mTOR mediated autophagy and intracellular ROS level. Biotechnol. Lett. 42 (4), 657–668. 10.1007/s10529-020-02818-z 31989342

[B9] Chitapanarux I. Tovanabutra N. Chiewchanvit S. Sripan P. Chumachote A. Nobnop W. (2019). Emulsion of olive oil and calcium hydroxide for the prevention of radiation dermatitis in hypofractionation post-mastectomy radiotherapy: A randomized controlled trial. Breast Care (Basel) 14 (6), 394–400. 10.1159/000496062 31933586 PMC6940468

[B10] Choi J. Moon S. Bae H. Kim Y. W. Seo Y. Wang H. S. (2020). Anti-inflammatory effects of alnus sibirica extract on *in vitro* and *in vivo* models. Molecules 25 (6), E1418. 10.3390/molecules25061418 32244969 PMC7145316

[B11] Deng X. Feng C. (2006). Clinical observation and nursing of fresh aloe vera juice for protecting radiation dermatitis. J. Pract. Med. 22 (10), 1211. 10.3969/j.issn.1006-5725.2006.10.055

[B12] Fallah M. Shen Y. Broden J. Backman A. Lundskog B. Johansson M. (2018). Plasminogen activation is required for the development of radiation-induced dermatitis. Cell Death Dis. 9 (11), 1051. 10.1038/s41419-018-1106-8 30323258 PMC6189099

[B13] Finberg M. J. Muntingh G. L. van Rensburg C. E. (2015). A comparison of the leaf gel extracts of Aloe ferox and Aloe vera in the topical treatment of atopic dermatitis in Balb/c mice. Inflammopharmacology 23 (6), 337–341. 10.1007/s10787-015-0251-2 26510768

[B14] Ghosh S. Ghosh A. (2021). Activation of DNA damage response signaling in mammalian cells by ionizing radiation. Free Radic. Res. 55 (5), 581–594. 10.1080/10715762.2021.1876853 33455476

[B15] Goyal P. K. Gehlot P. (2009). Radioprotective effects of Aloe vera leaf extract on Swiss albino mice against whole-body gamma irradiation. J. Environ. Pathol. Toxicol. Oncol. 28 (1), 53–61. 10.1615/jenvironpatholtoxicoloncol.v28.i1.60 19392655

[B16] Haddad P. Amouzgar-Hashemi F. Samsami S. Chinichian S. Oghabian M. A. (2013). Aloe vera for prevention of radiation-induced dermatitis: a self-controlled clinical trial. Curr. Oncol. 20 (4), e345–e348. 10.3747/co.20.1356 23904773 PMC3728063

[B17] Hong J. Wang H. Liu R. (2009). Analysis of curative effect of aloe vera in preventing and treating radiodermatitis in 50 cases. Pract. Clin. J. Integr. Traditional Chin. West. Med. 9 (1), 48–49.

[B18] Hong S. W. Chun J. Park S. Lee H. J. Im J. P. Kim J. S. (2018). Aloe vera is effective and safe in short-term treatment of irritable bowel syndrome: A systematic review and meta-analysis. J. Neurogastroenterol. Motil. 24 (4), 528–535. 10.5056/jnm18077 30153721 PMC6175553

[B19] Hoopfer D. Holloway C. Gabos Z. Alidrisi M. Chafe S. Krause B. (2015). Three-arm randomized phase III trial: Quality aloe and placebo cream versus powder as skin treatment during breast cancer radiation therapy. Clin. Breast Cancer. 15 (3), 181–190. 10.1016/j.clbc.2014.12.006 25619686

[B20] Kasmann L. Eze C. Taugner J. Roengvoraphoj O. Dantes M. Schmidt-Hegemann N. S. (2020). Chemoradioimmunotherapy of inoperable stage III non-small cell lung cancer: Immunological rationale and current clinical trials establishing a novel multimodal strategy. Radiat. Oncol. 15 (1), 167. 10.1186/s13014-020-01595-3 32646443 PMC7350600

[B21] Kma L. Baruah T. J. (2022). The interplay of ROS and the PI3K/Akt pathway in autophagy regulation. Biotechnol. Appl. Biochem. 69 (1), 248–264. 10.1002/bab.2104 33442914

[B22] Kong M. Hwang D. S. Lee J. Y. Yoon S. W. (2016). The efficacy and safety of Jaungo, a traditional medicinal ointment, in preventing radiation dermatitis in patients with breast cancer: A prospective, single-blinded, randomized pilot study. Evid. Based. Complement. Altern. Med. 2016, 9481413. 10.1155/2016/9481413 PMC4811089 27066103

[B23] Liao Y. Feng G. Dai T. Long F. Tang J. Pu Y. (2019). Randomized, self-controlled, prospective assessment of the efficacy of mometasone furoate local application in reducing acute radiation dermatitis in patients with head and neck squamous cell carcinomas. Med. Baltim. 98 (52), e18230. 10.1097/MD.0000000000018230 PMC6946454 31876704

[B24] Liu X. Chen Q. Lu Q. (2006). Observation on the effect of preventing and treating radiation skin damage with aloe juice. Shaanxi Med. J. 35 (10), 12511259–12521252. 10.3969/j.issn.1000-7377.2006.10.006

[B25] Liu X. (2007). Curative effect observation and nursing of aloe vera gel in preventing radiation dermatitis. Heilongjiang Med. J. 31 (10), 787. 10.3969/j.issn.1004-5775.2007.10.030

[B26] Merchant T. E. Bosley C. Smith J. Baratti P. Pritchard D. Davis T. (2007). A phase III trial comparing an anionic phospholipid-based cream and aloe vera-based gel in the prevention of radiation dermatitis in pediatric patients. Radiat. Oncol. 2, 45. 10.1186/1748-717X-2-45 18093332 PMC2238757

[B27] Mortezaee K. Goradel N. H. Amini P. Shabeeb D. Musa A. E. Najafi M. (2019). NADPH oxidase as a target for modulation of radiation response; implications to carcinogenesis and radiotherapy. Curr. Mol. Pharmacol. 12 (1), 50–60. 10.2174/1874467211666181010154709 30318012

[B29] Popanda O. Ebbeler R. Twardella D. Helmbold I. Gotzes F. Schmezer P. (2003). Radiation-induced DNA damage and repair in lymphocytes from breast cancer patients and their correlation with acute skin reactions to radiotherapy. Int. J. Radiat. Oncol. Biol. Phys. 55 (5), 1216–1225. 10.1016/s0360-3016(02)04415-2 12654430

[B30] Ramos-Tovar E. Muriel P. (2020). Free radicals, antioxidants, nuclear factor-E2-related factor-2 and liver damage. J. Appl. Toxicol. 40 (1), 151–168. 10.1002/jat.3880 31389060

[B31] Rao S. Hegde S. K. Baliga-Rao M. P. Palatty P. L. George T. Baliga M. S. (2017). An aloe vera-based cosmeceutical cream delays and mitigates ionizing radiation-induced dermatitis in head and neck cancer patients undergoing curative radiotherapy: A clinical study. Med. (Basel) 4 (3), E44. 10.3390/medicines4030044 PMC5622379 28930258

[B32] Richardson J. Smith J. E. Mcintyre M. Thomas R. Pilkington K. (2005). Aloe vera for preventing radiation-induced skin reactions: a systematic literature review. Clin. Oncol. 17 (6), 478–484. 10.1016/j.clon.2005.04.013 16149293

[B33] Rong X. Li J. Yang Y. Shi L. Jiang T. (2019). Human fetal skin-derived stem cell secretome enhances radiation-induced skin injury therapeutic effects by promoting angiogenesis. Stem Cell Res. Ther. 10 (1), 383. 10.1186/s13287-019-1456-x 31843019 PMC6916022

[B34] Sheng X. Zhou Y. Wang H. Shen Y. Liao Q. Rao Z. (2019). Establishment and characterization of a radiation-induced dermatitis rat model. J. Cell. Mol. Med. 23 (5), 3178–3189. 10.1111/jcmm.14174 30821089 PMC6484338

[B35] Shi G. Jiang H. Feng J. Zheng X. Zhang D. Jiang C. (2021). Aloe vera mitigates dextran sulfate sodium-induced rat ulcerative colitis by potentiating colon mucus barrier. J. Ethnopharmacol. 279, 114108. 10.1016/j.jep.2021.114108 33839199

[B36] Skalska-Kaminska A. Wozniak A. Paduch R. Kocjan R. Rejdak R. (2014). Herbal preparation extract for skin after radiotherapy treatment. Part One--Preclinical tests. Acta Pol. Pharm. 71 (5), 781–788. 25362806

[B37] Soria B. Martin-Montalvo A. Aguilera Y. Mellado-Damas N. Lopez-Beas J. Herrera-Herrera I. (2019). Human mesenchymal stem cells prevent neurological complications of radiotherapy. Front. Cell. Neurosci. 13, 204. 10.3389/fncel.2019.00204 31156392 PMC6532528

[B38] Su L. Wang Z. Huang F. Lan R. Chen X. Han D. (2018). 18β-Glycyrrhetinic acid mitigates radiation-induced skin damage via NADPH oxidase/ROS/p38MAPK and NF-κB pathways. Environ. Toxicol. Pharmacol. 60, 82–90. 10.1016/j.etap.2018.04.012 29677640

[B39] Surjushe A. Vasani R. Saple D. G. (2008). Aloe vera: a short review. Indian J. dermatol. 53 (4), 163–166. 10.4103/0019-5154.44785 19882025 PMC2763764

[B40] Tang W. Yang Z. He L. Deng L. Fathi P. Zhu S. (2021). A hybrid semiconducting organosilica-based O2 nanoeconomizer for on-demand synergistic photothermally boosted radiotherapy. Nat. Commun. 12 (1), 523. 10.1038/s41467-020-20860-3 33483518 PMC7822893

[B41] Tarameshloo M. Norouzian M. Zarein-Dolab S. Dadpay M. Mohsenifar J. Gazor R. (2012). Aloe vera gel and thyroid hormone cream may improve wound healing in Wistar rats. Anat. Cell Biol. 45 (3), 170–177. 10.5115/acb.2012.45.3.170 23094205 PMC3472143

[B42] Umehara Y. Toyama S. Tominaga M. Matsuda H. Takahashi N. Kamata Y. (2020). Robust induction of neural crest cells to derive peripheral sensory neurons from human induced pluripotent stem cells. Sci. Rep. 10 (1), 4360. 10.1038/s41598-020-60036-z 32152328 PMC7063040

[B43] Wang H. Li X. Liang W. (2018). Observation on the effect of fresh aloe vera in preventing breast cancer radiation dermatitis. World Latest Med. Inf. 18 (88), 202–206. 10.19613/j.cnki.1671-3141.2018.88.094

[B45] Wang K. X. Cui W. W. Yang X. Tao A. B. Lan T. Li T. S. (2021). Mesenchymal stem cells for mitigating radiotherapy side effects. Cells 10 (2), 294. 10.3390/cells10020294 33535574 PMC7912747

[B46] Williams M. S. Burk M. Loprinzi C. L. Hill M. Schomberg P. J. Nearhood K. (1996). Phase III double-blind evaluation of an aloe vera gel as a prophylactic agent for radiation-induced skin toxicity. Int. J. Radiat. Oncol. Biol. Phys. 36 (2), 345–349. 10.1016/s0360-3016(96)00320-3 8892458

[B47] Wilson A. Menon V. Khan Z. Alam A. Litovchick L. Yakovlev V. (2019). Nitric oxide-donor/PARP-inhibitor combination: A new approach for sensitization to ionizing radiation. Redox Biol. 24, 101169. 10.1016/j.redox.2019.101169 30889466 PMC6423503

[B48] Woo T. E. Somayaji R. Haber R. M. Parsons L. (2019). Scratching the surface: A review of dermatitis. Adv. Skin. Wound Care. 32 (12), 542–549. 10.1097/01.ASW.0000604184.92824.43 31764144

[B49] Wu M. Dong H. (2009). Observation on the effect of aloe vera gel in preventing radiation dermatitis. J. Guangdong Med. Coll. 27 (5), 554–555. 10.3969/j.issn.1005-4057.2009.05.032

[B50] Wu Y. Song Q. Miao H. (2015). Observation on the effect of aloe vera juice in preventing radiation dermatitis. Med. Inf. (12), 279. 10.3969/j.issn.1006-1959.2015.12.427

[B51] Yang L. Yu M. W. Wang X. M. Zhang Y. Yang G. W. Luo X. Q. (2016). Heijiangdan ointment relieves oxidative stress from radiation dermatitis induced by (60)Co gamma-ray in mice. Chin. J. Integr. Med. 22 (2), 110–115. 10.1007/s11655-015-2152-z 26142339

[B52] Yao H. Zou L. Wang M. (2006). Effect of applying aloe to prevent radiation injury of patients with skin malignancy. Chin. J. Nurs. 41 (4), 364–365.

[B53] Yew Y. W. Kuan A. George P. P. Zhao X. Tan S. H. (2022). Prevalence and burden of skin diseases among the elderly in Singapore: a 15-year clinical cohort study. J. Eur. Acad. Dermatol. Venereol. 36, 1648–1659. 10.1111/jdv.18205 35535625

[B56] Zhao Z. Fu X. Cai W. (2018). Intervention effect of different methods on radiodermatitis in patients with nasopharyngeal carcinoma undergoing radiotherapy. Today Nurse 25 (7), 100–102.

[B54] Zhang H. Jiang T. Mu M. Zhao Z. Yin X. Cai Z. (2022). Radiotherapy in the management of gastrointestinal stromal tumors: A systematic review. Cancers (Basel) 14 (13), 3169. 10.3390/cancers14133169 35804945 PMC9265110

[B55] Zhong Y. Yang Q. Chen X. (2017). Effect of ice compressing combined with external application of aloe on the prevention of nasopharyngeal carcinoma radioactive dermatitis. Nurs. Pract. Res. 14 (1), 139–141. 10.3969/j.issn.1672-9676.2017.01.059

